# Biochemical and Computational Analysis Of LNX1 Interacting Proteins

**DOI:** 10.1371/journal.pone.0026248

**Published:** 2011-11-08

**Authors:** Cheryl D. Wolting, Emily K. Griffiths, Renu Sarao, Brittany C. Prevost, Leanne E. Wybenga-Groot, C. Jane McGlade

**Affiliations:** 1 Department of Medical Biophysics, University of Toronto, Toronto, Canada; 2 Arthur and Sonia Labatt Brain Tumour Research Centre, Hospital for Sick Children, Toronto, Canada; University of Delhi, India

## Abstract

PDZ (Post-synaptic density, 95 kDa, Discs large, Zona Occludens-1) domains are protein interaction domains that bind to the carboxy-terminal amino acids of binding partners, heterodimerize with other PDZ domains, and also bind phosphoinositides. PDZ domain containing proteins are frequently involved in the assembly of multi-protein complexes and clustering of transmembrane proteins. LNX1 (Ligand of Numb, protein X 1) is a RING (Really Interesting New Gene) domain-containing E3 ubiquitin ligase that also includes four PDZ domains suggesting it functions as a scaffold for a multi-protein complex. Here we use a human protein array to identify direct LNX1 PDZ domain binding partners. Screening of 8,000 human proteins with isolated PDZ domains identified 53 potential LNX1 binding partners. We combined this set with LNX1 interacting proteins identified by other methods to assemble a list of 220 LNX1 interacting proteins. Bioinformatic analysis of this protein list was used to select interactions of interest for future studies. Using this approach we identify and confirm six novel LNX1 binding partners: KCNA4, PAK6, PLEKHG5, PKC-alpha1, TYK2 and PBK, and suggest that LNX1 functions as a signalling scaffold.

## Introduction

PDZ domains are protein interaction domains often found in molecules involved in signal transduction, cell polarity and synaptic transmission. PDZ domains were first found to recognize the extreme carboxy-terminal tail of target molecules by recognizing the last few amino acids as well as the terminal carboxylate residue [1]. PDZ domains have since been found to recognize other PDZ domains, internal sequences within targets, and phosphoinositides [2]–[4]. Proteins that contain PDZ domains often have multiple copies with distinct binding specificities and function as scaffolds in multiprotein complexes [5], [6].

LNX1 and LNX2 are RING finger domain-containing E3 ubiquitin ligases that each contain four PDZ domains [7]. Murine *Lnx1* mRNA is expressed in brain, heart, lung, skeletal muscle and kidney [8]. Human *LNX1* mRNA is similarly expressed in brain, heart, kidney as well as placenta and pancreas [9]. Murine *Lnx2* is more widely expressed and mRNA is detected in liver, spleen, lung, heart, kidney, thymus, skeletal muscle and brain [10]. In a pair-wise yeast two-hybrid assay, LNX1 has been shown to be able to interact with itself and LNX2 [10].

**Figure 1:**
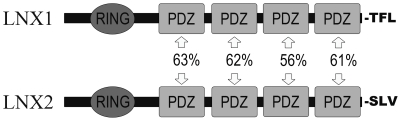
Schematic diagram of protein domains and carboxy terminal residues of LNX1 and LNX2. Percentages represent amino acid sequence identity between corresponding PDZ domains.

LNX1 was first identified as a binding partner of the cell-fate determinant Numb. The Numb phosphotyrosine binding (PTB) domain binds specifically to an NPXY motif found between the RING domain and the first PDZ domain (Figure 1) [8]. LNX1 mediates ubiquitination of Numb *in vitro* and targets specific Numb isoforms for degradation *in vivo* [7]. The four mammalian Numb isoforms differ by the presence or absence of two inserts, one in the amino terminal PTB domain and the other in the central proline rich region (PRR) [11]. LNX1 preferentially ubiquitinates the p72 and p66 isoforms, which contain the insert in the PTB domain, and a unique PDZ-PTB domain interaction contributes to the recognition of Numb for ubiquitination and degradation. [12].

The other PDZ domains of LNX1 have been shown to bind the cytoplasmic tail of transmembrane proteins including CAR (Coxsackievirus and Adenovirus Receptor), a tight junction protein that is important for viral binding to target cells [13]–[16], and Junctional Adhesion Molecule 4 (JAM4), an immunoglobulin superfamily member that is found at tight junctions [17], [18] and is redistributed during epithelial-to-mesenchymal transition [19], [20]. As well, the tight junction transmembrane protein, Claudin-1, has also been shown to interact through its carboxy-terminus with a region of LNX1 that includes all four PDZ domains [21]. In addition, LNX1 also binds ErbB2, a member of the Epidermal Growth Factor (EGF) Receptor family [22]. The second PDZ domain of LNX1 mediates the interactions with ErbB2, CAR and JAM4 [16], [20], [22]. Thus, LNX1 PDZ domains recognize the carboxy termini of several different transmembrane proteins.

A number of cytosolic proteins have also been identified as LNX1 PDZ binding proteins. A yeast two-hybrid screen to identify molecular partners of the pre-synaptic active zone protein CAST identified LNX1 [23]–[25]. The second PDZ domain of LNX1 was found to recognize the CAST carboxy terminus [25]. A yeast two-hybrid screen with LNX1 PDZ1 identified the transcription and splicing factor SKIP (Ski-interacting protein) [26]. In addition, a screen for binding partners for the potential PDZ recognition motif found in the proto-oncogene Src identified LNX1 PDZ3 [27]. *In vitro*, both PDZ1 and PDZ3 were able to interact with Src [27]. Recently a yeast two-hybrid screen for interaction partners of the small GTPase, RhoC, identified LNX1 and the PDZ1 domain was shown to be necessary for efficient binding to RhoC [28]. Finally, a yeast two-hybrid screen for Np9 interacting proteins identified three PDZ domain containing proteins, one of which was LNX1 [29]. Together, these individual studies demonstrate that LNX1 PDZ domains have distinct transmembrane and cytoplasmic binding partners and suggest that LNX1 may function as a molecular scaffold.

The first PDZ domain of LNX1 is known to recognize four cytoplasmic proteins, Numb, SKIP, RhoC and Src, two of which (Numb and Src) have also been identified as LNX1 ubiquitination substrates [7], [27]. The second PDZ domain of LNX1 is able to recognize the carboxy terminal tail of several different transmembrane proteins as well as one cytosolic protein, CAST. Thus far, Src is the only protein identified that interacts with the third PDZ domain of LNX1 and no interactions have been shown to involve the fourth PDZ domain of LNX1. Despite the number of potential protein-protein interactions identified for LNX1 to date, the lack of functional interrelationships between these interactors means that the cellular role of LNX1 is not well understood.

The arrangement of modular domains in LNX1 and their ability to recognize a wide variety of target proteins suggest that it acts as a scaffold molecule for a multiprotein signalling complex. In order to further elucidate the activities of LNX1 in the cell, we performed a protein array screen using the individual PDZ domains of LNX1 and identified 53 LNX1 interactors. These potential interactors were combined with the LNX1 interactors described above and predicted interactions mined from other high-throughput screens. Further bioinformatic and experimental analyses suggest that LNX1 functions as a membrane targeted scaffold protein involved in the assembly of signalling complexes regulated by ubiquitylation.

## Materials and Methods

### Expression and Purification of GST Fusion Proteins

The individual PDZ domains of hLNX1 were subcloned by PCR to create pEXP15-hLNX1 PDZ1 (aa 222–380), pEXP15-hLNX1 PDZ2 (aa 373–466), pEXP15-hLNX1 PDZ3 (aa 498–597), and pEXP15-hLNX1 PDZ4Δ3C (with last 3 amino acids deleted to remove a PDZ binding motif) (aa 630–725). Each pEXP15-hLNX1 PDZ construct was transformed into BL21-AI cells (Invitrogen, Carlsbad, CA) and induced to express GST fusion protein by the addition of 0.2% arabinose. Optimal expression was found under the following induction conditions: for pEXP15-hLNX1 PDZ1 3 h at 30°C; for pEXP15-hLNX1 PDZ2 3 h at 37°C, for pEXP15-hLNX1 PDZ3 and pEXP15-hLNX1 PDZ4Δ3C 1 h at 30°C followed by overnight at 4°C (no shaking). After induction, cells were pelleted and resuspended in PBS++++ (phosphate buffered saline, 1% Triton-X-100, 1% Tween-20, 1 mM dithiothreitol, Complete® EDTA-free protease inhibitor cocktail tablets (Roche Diagnostics, Indianapolis, IN)) at 10 mL of buffer/50 mL of culture. Resuspended cells were sonicated three times at 4°C for 30 s using 1 s on/1 s off pulsing. Sonicated cells were centrifuged for 10 minutes at 10,000 rpm in a Beckmann centrifuge rotor JA25.5. Supernatant was incubated with glutathione sepharose beads for 30–60 minutes at 4°C with rotation. Beads were washed 5 times with PBS++++. After washing, beads were resuspended in an equal volume of PBS++++.

As needed, GST fusion proteins were eluted from glutathione sepharose beads using 3 incubations with gentle mixing at 4°C with 10 mM glutathione in 50 mM Tris buffer pH 8.0. The concentration of fusion protein in each elution was estimated by running a 10 uL sample in 12.5% SDS-PAGE and comparing to known quantities of bovine serum albumin (BSA). Elutions containing sufficient amounts of protein were combined and dialysed overnight at 4°C against 1× PBS using Thermo Scientific Dialysis Cassettes (Slide-a-lyzers) with 10,000 molecular weight cut-off (Rockford, IL). The final concentration of eluted, dialyzed fusion protein was estimated by running a 10 uL sample in 12.5% SDS-PAGE and comparison to known quantities of BSA.

### Fluorescent Labelling of Purified Fusion Proteins

The Molecular Probes MP30009 Alexa Fluor 647 Microscale Protein Labelling Kit from Invitrogen (Carlsbad, CA) was used to fluorescently label approximately 20 ug of eluted and dialyzed fusion proteins GST-hLNX1 PDZ2, GST-hLNX1 PDZ3 and GST-hLNX1-PDZ4Δ3C according to the manufacturer's instructions (Figure S1). The amount of GST-hLNX1 PDZ1 produced was insufficient for labeling with Alexa-647.

### Protein Array Screens

In order to confirm that LNX1 PDZ domains were able to recognize known interactors in a protein array format, custom arrays were printed and screened. Regions of Numb (Entrez Gene ID 18222), either containing (PTB+) or lacking (PTB−) an 11 amino acid insert in the PTB region, were utilized. GST, GST-Numb PTB+ and GST-Numb PTB- were printed in rows at the following concentrations (ng/uL): 250, 125, 63, 32, 16, 8, 4. Each row also contained a blank spot identified as 0 ng/uL. This pattern of immobilized fusion proteins was repeated to create 12 sub-arrays per slide. We tested 0.05 ug, 0.10 ug and 0.20 ug of GST-hLNX1 PDZ2, GST-hLNX1 PDZ3 and GST-hLNX1 PDZ4Δ3C each individually and a combination of 0.05 ug, 0.10 ug and 0.20 ug of each of GST-hLNX1 PDZ2, GST-hLNX1 PDZ3 and GST-hLNX1 PDZ4Δ3C mixed together each on their own sub-array.

Optimized conditions were used to incubate the mixture of GST-hLNX1 PDZ2, PDZ3 and PDZ4Δ3C with duplicate Invitrogen human ProtoArray® v4 slides. Briefly, slides were rinsed with PBS+0.1% Tween 20 then blocked with blocking buffer (PBS, 1% BSA, 1 mM DTT, 0.1% Tween 20) for 2 hours at room temperature. Then 750 uL of reaction mixture (PBS, 1% BSA, 1 mM DTT, 5 mM MgCl_2_, 1% Triton-X-100, 1% glycerol, 0.33 ug/mL Alexa647-labelled hLNX1 PDZ2, 0.50 ug/mL Alexa647-labelled hLNX1 PDZ3, 0.20 ug/mL Alexa647-labelled hLNX1 PDZ4Δ3C) was pipetted over slides and incubated for 90 minutes at room temperature. Slides were washed three times for 15 minutes each time with wash buffer (PBS, 1 mM DTT, 5 mM MgCl_2_, 0.5% Triton-X-100). Slides were left to dry overnight in the dark. Fluorescence was visualized using a PE ProScanArrayHT with 633 nm laser at 10 uM resolution. Spot fluorescence was corrected for local background and normalized using LOWESS normalization as available in the ProScanArray Express software (Perkin Elmer). Quantitated spot fluorescence was adjusted for the concentration of protein at each spot using ProtoArray Prospector® (Invitrogen). Every protein is spotted twice on the human ProtoArray® v4. A protein was considered a hit on a given slide if both spots have an average Z-score of greater than three and the coefficient of variation for the signals from the two replicates is less than 0.5.

### Yeast Two-Hybrid Screen

Expression of GAL4-DNA binding domain-mLNX fusion protein in L40 yeast cells (MATa, his3D200, trp1–901, leu2–3,112, ade2, LYS2::(lexAop)_4_-HIS3 URA3::(lexAop)_8_-lacZ) was confirmed by western blot of transformed yeast cell lysate. The yeast two-hybrid screen was performed as previously described [8]. Prey clones containing insert were selected for maxiprep (Qiagen), sequenced and retransformed with pBTM116-mLNX to confirm the interaction and determine specificity. Sequences were analysed by BLAST and FASTA searches of GenBank. Matching or similar sequences were verified with the PILEUP and BESTFIT programs from the GCG Wisconsin package.

### Identification of Additional LNX1 Interacting Proteins

We assembled a list of other LNX1 interactors by searching published literature and interaction databases (GRID [30], UniHI [31], Domino [32], and PDZbase [33]). The list of published and potential LNX1 interactors contains 220 mammalian proteins.

### Clustering by Gene Ontology Annotation

The set of 220 mammalian LNX1 interactors were clustered by Gene Ontology Annotation (GOA) as previously described [34]. Briefly, the similarity scores (ranging from 0.0–1.0) for all pairs of proteins in the set were calculated using the simUI method in R/BioConductor and converted to dissimilarity scores (1.0-similarity). The matrix of dissimilarity scores was used as input for Partitioning Around Medoids (PAM) clustering in R/BioConductor. The R/BioConductor method silcheck was used to select k, the number of clusters. The resulting clusters were visualized in Cytoscape [35], where nodes represent proteins and edges represent dissimilarity between proteins. As every protein in the list is compared to every other protein in the list, an edge exists between every pair of nodes. In order to improve visualization, edges representing dissimilarity scores above 0.4 (BP, MF) or 0.3 (CC) were removed and dissimilarity scores close to 0 are shown as darker, thicker edges. Circles of nodes represent proteins that were clustered together. Each circle of nodes contains one larger node that represents the medoid (i.e., the representative object) for that cluster. The label for each cluster is a term selected from the set of GO terms assigned to the medoid protein and found by manual inspection to be representative of the GOA of the majority of the remaining proteins in the cluster. The colour of each node was randomly assigned by Cytoscape according to the GOA from the corresponding GO aspect (BP, MF or CC).

### Plasmids

Murine LNX1, LNX1C45A [11], LNX2, LNX2C51A were cloned into pFLAG-CMV-2. Full length human AURKB, CDK2, CAMK2N2, KCNA4, MAPKAP3, PLEKHG5, RASL11B, ARVCF, PKC-alpha1, PAK6, and TYK2 and were cloned in Gateway N-terminally tagged destination vector N-Myc.

### Transfections and Co-immunoprecipitations

293T cells were maintained in 10% FBS in DMEM (Dulbecco's Modified Eagle Medium). Cells were transfected with 2 µg of each plasmid and transfected at 50–60% confluency with 5–10 ug of pFLAG-LNX1, pFLAG-LNX2 with or without N-myc tagged cDNAs as described above, using OPTI-MEM and lipofectamine 2000 (GibcoBRL). After 24 hours, cells were lysed with HNTG-ZE [11] containing protease inhibitors (Complete). 1 mg of total protein was precleared with protein-G sepharose for 30 min and then immunoprecipitated with 1 µg of anti-Myc antibody (9E10). The beads were boiled for 5 min in SDS Sample buffer and separated by SDS-PAGE, transferred to PVDF and immunoblotted with anti-Flag (M2, Sigma) or anti-Myc at 1∶1000.

### GST Fusion Protein Binding Experiments

Transfected 293T cell lysate (50–100 uL) was mixed with 3 ug of purified GST fusion protein immobilized on glutathione sepharose beads in a total of 1 mL PLC lysis buffer [8] for 90 min at 4°C. Sepharose beads were pelleted and washed five times with 1 mL PLC lysis buffer. The beads were resuspended in 70 uL of 2xSDS sample buffer with 5% ß-mercaptoethanol and boiled 5–7 min at 100°C. The samples were separated by 10% SDS-PAGE, transferred to immobilon-P and western blot analysis with an anti-myc antibody was performed to detect interactions.

### Lipid Binding Experiments

Binding of GST-LNX1 and individual GST-LNX PDZ domains to immobilized PIP lipids was performed according to manufacturer's protocol with some modifications. Briefly, PIP Strip membranes (Echelon Biosciences) were blocked with TBS containing 3% (w/v) Bovine Serum Albumin (BSA). Blocked membranes were incubated with GST-LNX1 (0.025 µg/ml) and GST-LNX1 PDZ domains (1 µg/ml) in TBS with 3% BSA, at 4°C overnight. Membranes were washed three times with TBS containing 0.1% Tween-20 and then incubated with rabbit anti-GST (2 µg/ml) in blocking solution for one hour at room temperature. After washing three times with TBS with 0.1% Tween-20, membranes were incubated with anti-rabbit HRP (Horseradish Peroxidase) in blocking solution, washed, developed using ECL (Enhanced Chemi-Luminescence, Amersham) and exposed to film.

## Results

### LNX1 PDZ Domains Are Most Similar to LNX2 PDZ Domains

A recent study demonstrated that the binding specificity for a given PDZ domain can be reliably predicted from that of another PDZ domain with known specificity if there exists sufficiently high identity in the binding site sequence, as represented by 17 residues that have contact with the ligand [36]. Specifically, binding site sequence identity over 70% between PDZ domains predicts that the two domains will have a similar binding specificity. In order to determine if the binding specificity of the LNX1 PDZ domains could be predicted, we performed BLAST searches of the NCBI GenPept protein sequence database to identify the most similar PDZ domains for each LNX1 PDZ domain. The strongest similarity as measured by percent sequence identity for each of the LNX1 PDZ domains was with the corresponding PDZ domain in LNX2 (sequence identity range: 56–63%) (data not shown). The next best hit for each PDZ domain had an average 19% lower similarity score (sequence identity range: 35–48%). We examined the sequence identity in the 17 residues identified as the binding site sequence between each LNX1 PDZ domain and the respective top three similar PDZ domains. PDZ1 and PDZ3 did not have binding site sequence identity over 70% with any of the PDZ domains examined. PDZ2 and PDZ4 had binding site sequence identity over 70% with only LNX2 PDZ2 and PDZ4, respectively. However, since the binding specificities for the LNX2 PDZ domains are not yet known, we were not able to predict the binding specificity for the LNX1 PDZ domains.

### Identification of LNX1 PDZ Binding Proteins Using a Protein Array Screen

In order to identify novel LNX1 PDZ domain binding targets we screened Invitrogen ProtoArray® v.4 slides with the isolated PDZ domains of LNX1 expressed as GST fusion proteins. We used custom printed protein arrays to optimize conditions for LNX1 PDZ domain binding to immobilized fusion proteins. Test protein arrays were printed with positive and negative control proteins to confirm that Alexa 647-labelled GST-LNX1 PDZ domains were able to bind to immobilized GST fusion proteins but not GST alone. The fusion proteins GST, GST- Numb PTB+ and GST-Numb PTB- were printed on PATH slides at eight concentrations ranging from 0–250 ng/uL. We found that the individual hLNX PDZ domains bound to proteins on the protein array with differing efficiency, therefore, different amounts of each were used in the screen. Specifically, GST-hLNX PDZ4Δ3C binding to positive controls was detectable at a lower concentration than binding by GST-hLNX PDZ3. Therefore, the final screen utilized 0.33 µg/mL GST-hLNX PDZ2, 0.50 µg/mL GST-hLNX PDZ3 and 0.20 µg/mL GST-hLNX PDZ4Δ3C.

**Figure 2:**
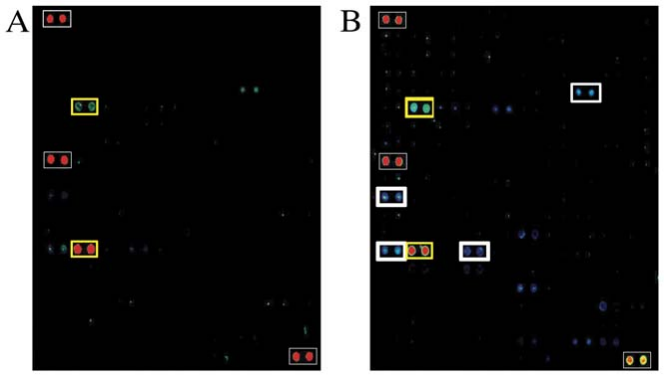
Two Invitrogen ProtoArrays® v4 were incubated with a mixture of the following Alexa647-fluorescently labelled proteins: 0.33 ug/mL LNX1 PDZ2, 0.5 ug/mL LNX1 PDZ3 and 0.2 ug/mL LNX1 PDZ4Δ3C. (A,B) A sample grid (grid #43) is shown from each of the two arrays. Reference spots for grid identification are outlined in thin white rectangles. Thick yellow rectangles indicate LNX1 interactors identified on both protein arrays: BC004233.1 28AA in row 6 columns 3 and 4 and NM_172160.1 KCNAB1 in row 14 columns 3 and 4. (B) Thick white rectangles indicate LNX1 interactors identified on only one protein array: NM_020646.1 ASCL3 in row 5 columns 15 and 16, NM_020548.4 DBI in row 11 columns 1 and 2, NM_153752.1 untranslated mRNA in row 14 columns 1 and 2, and NM_016096.1 ZNF706 in row 14 columns 7 and 8.

Duplicate Invitrogen ProtoArray® v.4 slides were treated identically and probed with a mixture containing Alexa 647-labelled GST-PDZ2, -PDZ3 and -PDZ4Δ3C (Figure 2). Slides were scanned and analyzed according to manufacturer instructions. Since each protein was spotted twice on each protein array, a protein was considered to interact with LNX1 if both spots met the cut-off criteria (see Experimental Procedures). Slide 1 yielded 123 proteins and slide 2 had 96 proteins that met our criteria for positive binding. Of these, 62 proteins were positive for LNX1-PDZ domain binding on both slides (Table S1).

Of the 62 proteins identified as LNX1 PDZ domain binding partners, four proteins corresponded to 3′ untranslated regions of cDNA, three proteins corresponded to cDNA that has been removed from NCBI's GenBank database due to standard genome annotation, one protein corresponded to a cDNA that is permanently suppressed in NCBI's GenBank database as it is now thought that this gene does not encode a protein and one protein corresponded to a cDNA that is permanently suppressed in NCBI's GenBank database as it is a non-sense mediated decay candidate. The 53 remaining proteins corresponded to annotated proteins.

As it has been shown that some PDZ domains are able to recognize and bind other PDZ domains, we searched the 53 hits for proteins containing PDZ domains. We found that only one protein, DEPDC6, that contains two DEP domains and one PDZ domain. The very low number of hits containing PDZ domains could indicate that LNX1 PDZ domains do not recognize other PDZ domains, that the LNX1 PDZ domains do not recognize other PDZ domains when expressed individually or that LNX1 PDZ domains are not able to effectively recognize other PDZ domains in this assay format.

Among the 53 newly identified LNX1 PDZ binding proteins, 11 proteins were found to contain a kinase domain: DAPK1, PAK1, CDK2, PLK3, AURKB, AURKC, MAPKAPK3, DYRK3, EPHA8, EPHB3 and TRIB1 (Table S1). To determine if the high number of kinase domain-containing proteins identified simply reflected a high proportion of kinases on the protein array, we searched the protein content list provided by Invitrogen for the ProtoArray® v.4. While 5.6% (448/8000) of the proteins on the array were annotated as Protein Kinases, 17.7% (11/62) of LNX1 hits contain protein kinase domains. We calculated the fraction of hits that are protein kinases using all hits (62) rather than only hits that correspond to annotated proteins (53) since the percentage of the 8000 proteins on the ProtoArray® v.4 that correspond to annotated proteins is unknown.

Inspection of the disease relationships of the 53 hits revealed 11 proteins that have previously been linked to tumours and/or cancer (Table S1). The proteins DDX17, MPG and PAK1 have been linked to colorectal cancer. Aberrant expression of AURKB, MPG and SNCB has been observed in cancers of neuronal cell types - gliomas, astrocytomas and medulloblastomas, respectively. AURKC is known to be over expressed in several cancer cell lines while DAPK1 is a tumour suppressor candidate. Finally, CDK2, LST1, NKD2 and RASL11B have also been implicated in a variety of cancer types.

We also identified three proteins, KCNA4 (Kv1.4), KCNAB1 and KCNAB2 that are voltage-gated potassium channel subunits while another hit, ARPP-19, is able to act as a potassium channel regulator. In addition, SCLT1 is known to be a sodium channel subunit. This is in keeping with the observation that a number of PDZ domain containing proteins are known to associate with and regulate activity of transmembrane proteins and channels. Finally, nine of the 53 hits are uncharacterized proteins containing either no known domains or domains of unknown function.

### Specificity of the LNX1 PDZ Domains

We examined the carboxy-terminal tails of all 62 proteins for information about the binding selectivity of the LNX1 PDZ domains. Approximately one-third of the hits (21) had carboxy terminal tails that conform to an expanded list of PDZ domain binding motifs based on Tonikian et al., [36] (Table 1). For 15 of these proteins, the carboxy terminus of the protein spotted on the ProtoArray® v.4 corresponds to the predicited natural carboxy terminus of the protein. Five of the 21 proteins (EphB3, DAPK1, AURKC, NKD2, and KIAA1598) had artificially created carboxy terminal tails as they were encoded by partial cDNAs, while one protein (EBF4) contained a potential PDZ recognition motif but does not correspond to an annotated protein. With respect to the amino acid at the carboxy terminus (0 position), three of the 21 proteins we identified contained a cysteine at that position. While not previously recognized as a canonical PDZ recognition motif, LNX1 PDZ2 has previously been reported to select peptides with a terminal cysteine residue [37], and more recently PDZ domains from *C. elegans* and humans have been shown to preferentially recognize motifs with a carboxy terminal cysteine [36]. The remaining 17 carboxy terminal sequences conform more closely with the major class of PDZ domain binding motif that have a hydrophobic residue at the 0 position.

**Table 1:** Carboxy terminal amino acids of selected ProtoArray LNX1 interacting proteins.

Protein	Proto-array C-term sequence	Wild-type C-term sequence
**AMMECR1L**	RSV RSR SPH ESV*	Not translated to protein
**AURKB**	RVL PPS ALQ SVA*	WT
**AURKC**	GRA DPA FLY KVV*	RRV LPP CAQ MAS*
**C1ORF183**	PSG FDI NTA VWV*	WT
**CAMK2N2**	LKG MGE KPP SGV*	WT
**DAPK1**	GRA DPA FLY KVV*	TSY NSI SSV VSR*
**DEPDC6**	TIV MEV MEE LEC*	WT
**EBF4**	SPA RGL QGL AYS*	Not translated to protein
**EPHB3**	GRA DPA FLY KVV*	RLQ MNQ TLP VQV*
**IMAGE:4829245**	DKA MLE LGP HIC*	WT
**KIAA1598**	IEN VRE TDS SNC*	AVD ELK GIL ASQ*
**MGC18299**	NKQ NYT KPT TSV*	WT
**MRPS24**	QTV PSK VVY KYL*	WT
**MUSTN1**	KPK AGP TKS VFG*	WT
**NKD2**	WPG PFP SGL VAV*	WT
**PLEKHG5**	LLL NST LTA SEV*	WT
**PPID**	DKE KAV YAK MFA*	WT
**RASL11B**	ALS AKV RTV TSV*	WT
**SCLT1**	GEL NGQ LKY YQA*	WT
**SNCB**	PQE EYQ EYE PEA*	WT
**ZADH2**	VVE LPH SVN SKL*	WT

To further investigate the binding specificity of LNX1 PDZ2 we superimposed the structure of LNX PDZ2 (PDB id: 2VWR) with that of PSD-95 PDZ3 bound to a Class I PDZ ligand (PDB id: 1TP3), and modeled mutagenesis of the ligand's carboxy terminal valine to a cysteine residue using Coot [38]. This analysis showed that a peptide with a carboxy terminal Cys residue could be accommodated in the binding pocket of both LNX PDZ2 and PSD95 PDZ3. Since the cysteine side chain is smaller than those typically present in position 0 of Class I PDZ domains, we measured the predicted distances between residues that form the hydrophobic binding pocket and the carboxy terminal Cys residue. We did not find any significant differences to suggest that an additional contact helps stabilize a Cys peptide interaction with LNX PDZ2 (data not shown). A search of the literature found four additional PDZ domains reported to bind carboxy terminal Cys peptides, from PDZ-RhoGEF, APBA3, SITAC and PTP-BL proteins [36], [39]–[41]. Using ClustalW, we compared the sequences of 29 type I PDZ domains classified by Tonikian et al. [36] with known Cys-peptide binding domains, as well as LNX1 PDZ2 and LNX2 PDZ2. This analysis revealed that PDZ domains reported to bind Cys-peptide are more closely related to each other than to other Class 1 PDZ domains, based on primary sequence (Figure S2). This suggests that regions other than the hydrophobic binding pocket may contribute to stabilizing the interaction with Cys containing peptides.

### Assembly of a Comprehensive List of LNX1 Interactions

In addition to the protein array screen, we performed a yeast two-hybrid screen with LNX1. Co-transformation of pBTM16-mLNX1 with 100 ug of a mouse embryo heart and lung library produced 1.5×10^6^ transformants. Of the 77 colonies that were selected and restreaked from the library plates, 44 clones exhibited ß-galactosidase activity. After eliminating false positives, out of frame fusions and fusions with non-coding regions, we found six clones that interacted with an individual LNX1 PDZ domain (Table 2).

**Table 2:** LNX1 interacting proteins from yeast two-hybrid screen.

Protein	Entrez Gene ID	Description	C-terminus
**WWP1**	11059	WW containing E3 ubiquitin ligase	FAIEETEGFGQE
**NG23**	401251	Chromosome 6 open reading frame 26	GCTKGPRGPTRV
**PRA1**	10567	RABAC1; prenylated Rab acceptor	AVDGEELQMEPV
**TP14C**	51522	transmembrane protein 14C	AKVGVSMFNRPH
**WAC**	51322	WW domain containing adaptor with a coiled coil region	LEKLKNQNSFMV
**Syntaxin5**	6811	syntaxin 5A	IVFFIIFVVFLA

LNX1 has been included in several high-throughput interaction screens identifying an additional 156 potential interaction partners. Rual et al. tested all pair wise interactions of 7194 proteins from the human ORFeome collection in a high-throughput yeast two-hybrid study [42]. LNX1 was used as both bait and prey and interacted with 43 proteins [38]. In a screen against a random peptide library in the yeast two-hybrid system, the profile of peptides recognized by LNX1 PDZ2 was used to identify 107 predicted LNX1 PDZ2 interactors [37]. Finally, in a screen of 157 mouse PDZ domains against 217 peptides based on the carboxy termini of mouse proteins, four proteins were identified as interactors for LNX1 PDZ2 [43]. None of the LNX1-interacting proteins were found in common between these studies, perhaps as a consequence of the different types of assays used in the three screens. However, two interactors, PRA1 and DDX17, were identified by both high-throughput yeast two-hybrid [42] and our studies. We isolated PRA1 in our yeast two-hybrid screen and DDX17 in our protein array screen. We used the combined results to generate a list of 220 mammalian LNX1 interacting proteins for further analysis (Table S2).

### Clustering LNX1 Interacting Proteins by Gene Ontology Annotation

**Figure 3:**
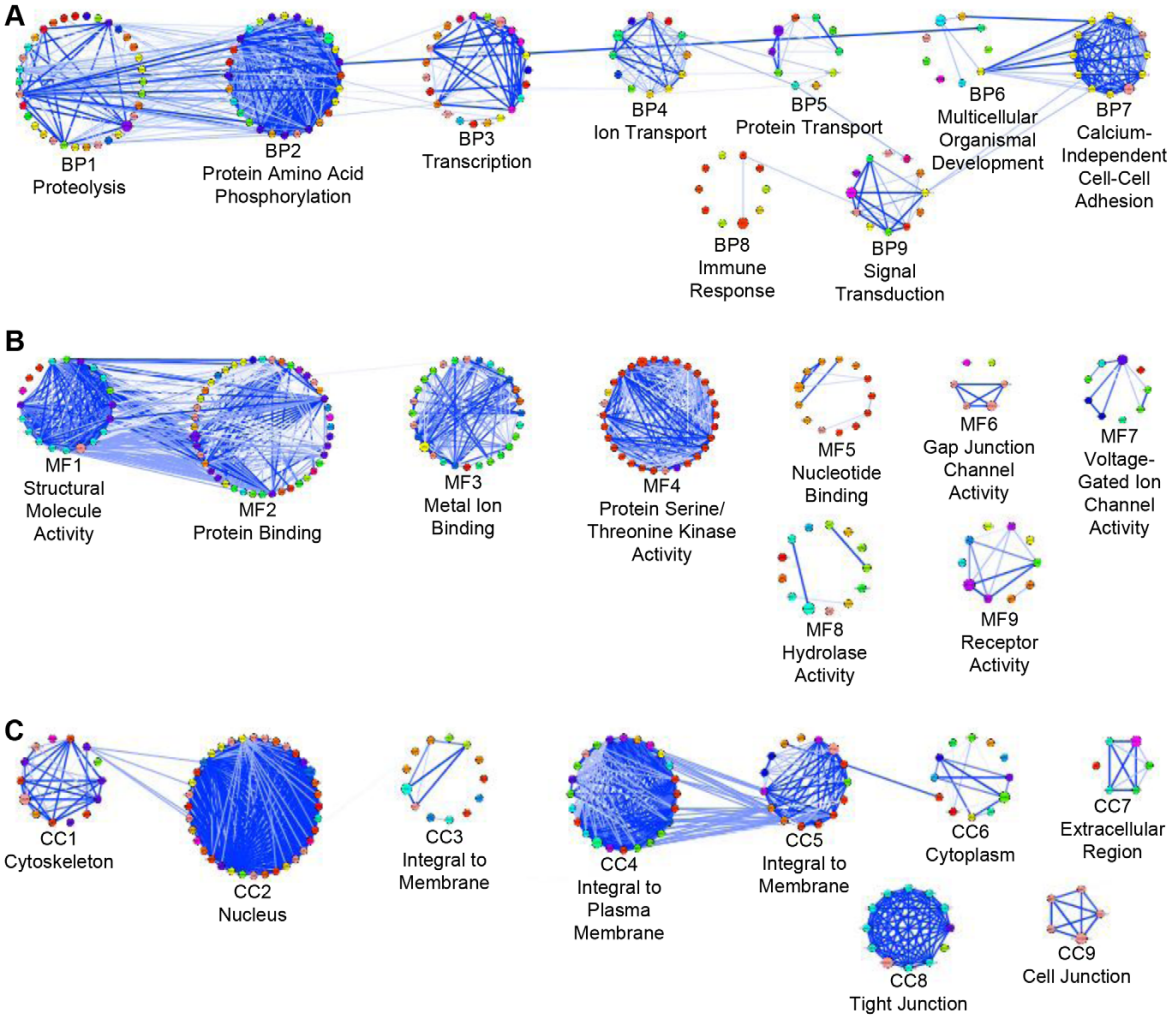
Clustering by GO annotation similarity. 220 LNX1 interacting proteins were clustered according to the graph similarity of their GO annotation. Nodes represent proteins; lines between nodes represent the similarity of GO annotation between two proteins with thick dark lines representing high similarity and thin light lines representing lower similarity scores. Node colours represent GO annotation and were randomly assigned by Cytoscape. Proteins are grouped together in circles according to their clusters, i.e. circle 1 = cluster 1. (A) Proteins clustered according to the similarity of their GO Biological Process annotation, clusters BP1–BP9. (B) Proteins clustered according to the similarity of their GO Molecular Function annotation, clusters MF1–MF9. (C) Proteins clustered according to the similarity of their GO Cellular Component annotation, clusters CC1–CC9.

In order to reveal potential new roles for LNX1, the list of putative LNX1 interacting proteins was clustered by associated GOA (Figure 3) [34]. The resulting clusters were visualized in Cytoscape; nodes represent proteins and edges represent the strength of the GO similarity between pairs of proteins. This analysis revealed well separated clusters, such as MF3, MF4, CC2 and CC8, which contain high similarity between the proteins within each cluster. These clusters contain proteins that are highly related to each other but not to proteins from other clusters. Several adjacent clusters such as BP1–BP2 and MF1–MF2 are not well separated and have many edges between two clusters in addition to those within the cluster. These clusters contain proteins that are closely related to proteins from two different clusters. This phenomenon has been observed in all clustering by GOA as performed using our method and is believed to reflect the current level of granularity of GO itself. As both GO and the number of proteins that have GOA grow, clustering by GOA is expected to produce better separated clusters.

### Validation of Novel LNX1 PDZ Binding Proteins

**Figure 4:**
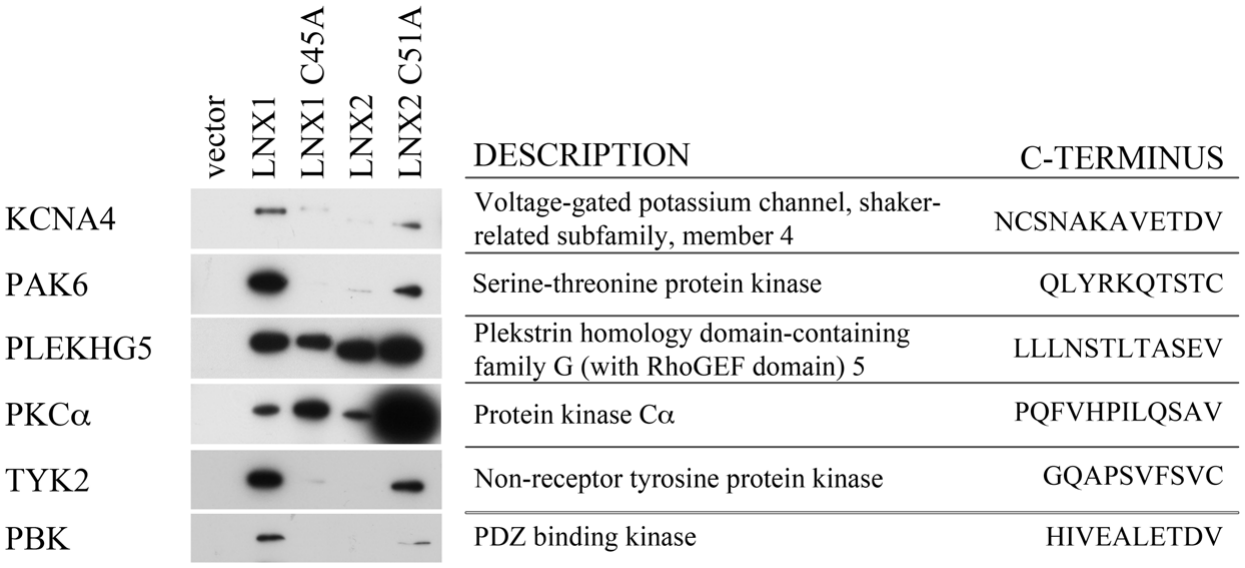
Confirmation of novel LNX1 and LNX2 binding partners by co-immunoprecipitation. Flag-tagged LNX1, LNX1 C45A (inactive ubiquitin ligase), LNX2 or LNX2 C51A (inactive ubiquitin ligase) were cotransfected with Myc-tagged candidates into 293T cells. Cell lysates were immunoprecipitated with anti-Myc antibody and blotted with anti-Flag to identify novel interactions.

Two clusters were selected as having GOA trends of biological interest: MF1 – Structural molecule activity (containing 20 proteins) and MF4–Serine/threonine kinase activity (containing 30 proteins). To determine whether interactions identified in the protein array and other high-throughput screens could be verified by co-immunoprecipitation, we obtained cDNAs corresponding to 14 proteins for further study. We selected three proteins from MF1, seven proteins from MF4 (protein serine/threonine kinase activity), as well as one protein from MF7 (voltage-gated ion channel activity), one protein from MF9 (receptor activity) and two proteins that did not have GOA (Table 3). These cDNAs were subcloned into a myc tagged mammalian expression vector and cotransfected with FLAG-tagged LNX1, LNX1C45A (inactive ubiquitin ligase), LNX2, LNX2 C51A (inactive ubiquitin ligase) or empty vector control. LNX1 family proteins were found to immunoprecipitate with KCNA4, PAK6, PLEKHG5, PKC-alpha1, TYK2, and PBK (Figure 4) while AURKB, CDK2, CAMK2N2, MAPKAPK3, Claudin1 and Claudin3 did not coimmunoprecipitate (data not shown). Four of the confirmed interacting proteins contain classic S/T- X-V binding motifs while the remaining two, PAK6 and TYK2, contain a novel class of PDZ binding motif with a cysteine in the 0 position (S-X-C) (Figure 4). Different patterns of target binding to LNX1, LNX2 and their respective ligase inactive forms, was observed in these experiments. The C45A mutation within the RING domain is thought to disrupt its interaction with E2 enzymes and we have shown that this mutation abolishes LNX1 ability to ubiquitinate Numb [7]. This might be expected to stabilize certain interactions in which the binding target is also a substrate. Alternatively, as this mutation disrupts interaction with E2 enzymes by unfolding the RING domain, it could also cause disruption in the folding of other nearby regions, including the PDZ domains, important for target binding.

**Table 3:** Proteins selected for testing by co-immunoprecipitiation.

Protein	Entrez Gene ID	Description	Carboxy-terminus	Source	MF cluster
**ARVCF**	421	armadillo repeat protein deleted in velocardiofacial syndrome	GDAKPQPVDSWV	MCP 2006 5:1368	1
**claudin-1**	9076	tight junctions between epithelial cells	PAPSSGKDYV	MCP 2006 5:1368	1
**Claudin-3**	1365	tight junction protein, low affinity receptor for Clostridium perfringens enterotoxin	GTGYDRKDYV	MCP 2006 5:1368	1
**AURKB**	9212	Aurora kinase B	RVLPPSALQSVA	ProtoArray screen	4
**CDK2**	1017	Cyclin-dependent kinase 2	QDVTKPVPHLRL	ProtoArray screen	4
**MAPKAPK3**	7867	Mitogen-activated protein kinase-activated protein kinase 3	GSSSASQGCNNQ	ProtoArray screen	4
**PAK6**	56924	serine/threonine-protein kinase	QLYRKQTSTC	MCP 2006 5:1368	4
**PBK**	55872	PDZ-binding kinase	HIVEALETDV	MCP 2006 5:1368	4
**TYK2**	7297	non-receptor tyrosine-protein kinase	GQAPSVFSVC	MCP 2006 5:1368	4
**PKC**	5578	Protein Kinase C alpha	PQFVHPILQSAV	Science 2007 317:364	4
**KCNA4**	3739	potassium channel Kv1.4; KCNA4; human NM_002233	NCSNAKAVETDV	MCP 2006 5:1368	7
**PLEKHG5**	57449	Pleckstrin homology domain containing family G (with RhoGEF domain) member 5	LLLNSTLTASEV	ProtoArray screen	9
**RASL11B**	65997	Ras-like family 11 member B	ALSAKVRTVTSV	ProtoArray screen	
**CAMK2N2**	94032	Calcium/calmodulin-dependent protein kinase II inhibitor 2	LKGMGEKPPSGV	ProtoArray screen	

**Figure 5:**
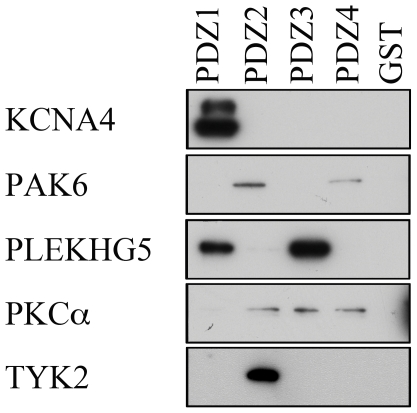
Interaction of individual LNX1 PDZ domains with novel binding partners as tested by fusion protein binding experiments. Each PDZ domain of LNX1 was fused to GST. Purified fusion proteins were incubated with 293T cell lysate transfected with the myc-tagged protein indicated at left. Interactions were detected by immunoblotting with anti-myc antibody.

We then investigated which of the LNX1 PDZ domains were able to recognize the coimmunoprecipitated proteins described above by performing GST-fusion pull downs with isolated LNX1 PDZ domains (Figure 5). GST-PDZ1 was able to bind KCNA4 and PLEKHG5. GST-PDZ2 recognized PKC-alpha1, TYK2, and PAK6. GST-PDZ3 bound only PLEKHG5 while GST-PDZ4 recognized PKC-alpha1 and PAK6 (Figure 5). PBK was not recognized by any individual LNX1 PDZ domains (data not shown).

Next we used the carboxy terminal residues of the proteins recognized by individual LNX1 PDZ domains to search the list of 220 LNX1 interactors for other proteins that have similar carboxy termini (Table S3). Allowing for conservative amino acid substitutions in the final three residues of the protein, 28 proteins were identified. Of the 28 proteins, 27 were predicted LNX1 interactors from the phage-display random peptide library screen [37] while the remaining protein was identified in our protein array screen.

### Investigation of LNX1 PDZ Domain Lipid Binding Specificity

**Figure 6:**
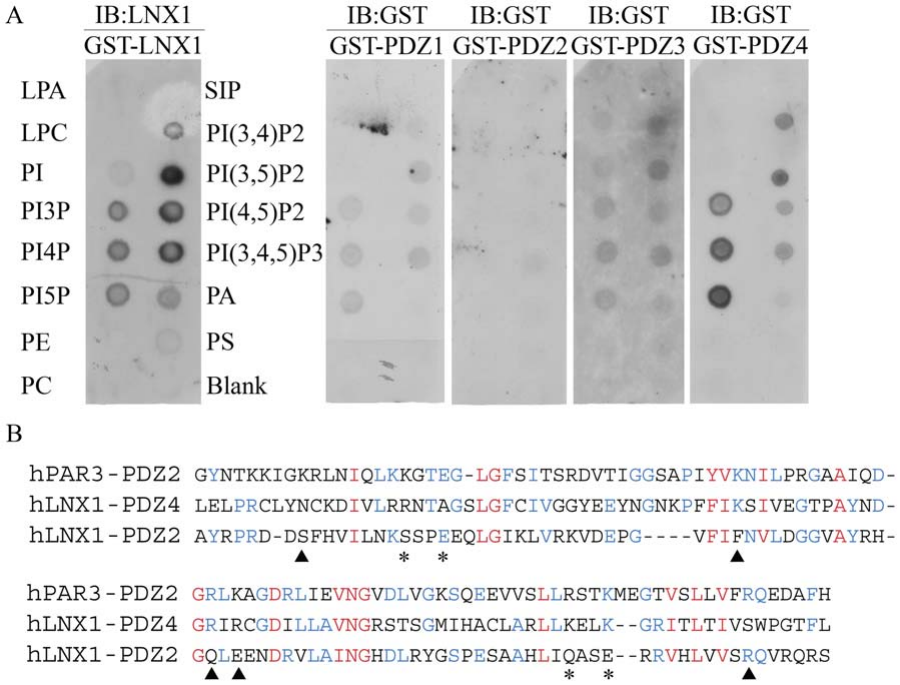
LNX1-lipid binding. (A) Binding of purified LNX1 or GST-LNX1 PDZ1, 2, 3 or 4 to phosphatidylinositol phosphate lipids immobilized on membrane strips (Echelon Biosciences) was assessed by anti-LNX1 or anti-GST immunoblot. Exposure times of individual membrane strips were: LNX1, 1 seconds.; PDZ1, 30 min.; PDZ2, 1 min.; PDZ3, 30 min., PDZ4, 15 seconds. (B) Sequence comparisons of LNX1 PDZ 2 and 4 and the lipid membrane-binding PDZ 2 of Par3. Residue positions shown to be critical for forming a positive charge cluster (▴) or the binding pocket for the phosphatidylinositol head group (*) in Par3 PDZ 2 are indicated.

As PDZ domains have also recently been shown to interact with membrane phosphoinositides (PI) [4], [44]–[46], we investigated the phospholipid binding of the individual LNX1 PDZ domains. Using membranes containing various immobilized phospholipids, we found that full length LNX1 and the isolated LNX1 PDZ4 bound specifically to PIPs but not to other phospholipids (Figure 6A). PDZ1 and PDZ3 also showed very weak binding to the same PIPs and PDZ2 did not bind detectably to any phospholipids on the membrane. In support of the observation that PDZ4 recognizes PIPs while PDZ2 does not, a sequence alignment of PDZ2 and PDZ4 with hPar3 PDZ2, a PDZ domain known to bind PIPs, revealed that many of the residues that form a positive charge cluster important for PIP binding by PAR3 are conserved in LNX1 PDZ4 but not in LNX1 PDZ2 (Figure 6B) [46].

## Discussion

In this study, protein array and yeast two-hybrid screens were performed to identify binding partners for the PDZ domains of the E3 ubiquitin ligase LNX1. We combined our data with that of published reports to develop a comprehensive list of potential LNX1 binding partners. Computational analysis of this large data set has further defined the LNX1 PDZ domain specificities, and cluster analysis based on Gene Ontology annotation has revealed potential new biological roles for LNX1.

In addition to the protein array screen performed here, two other published accounts used LNX1 as bait for high-throughput screens of defined libraries. In Rual et al. a high-throughput yeast two-hybrid screen, 7194 human Entrez Gene IDs were provided for the defined library [42]. In our protein array screen, there were 6740 molecules for which human Entrez Gene IDs could be identified. In Stiffler et al. a screen of immobilized peptides based on mouse proteins, human Entrez Gene IDs could be found for 181 of 217 molecules [43]. Of these, only one protein was found in common between any of the screen results. The low overlap between screen results is consistent with observations from other high-throughput screen comparisons [47], [48]. In part, this can be because the libraries screened did not contain the same target proteins or may reflect species divergence in the libraries used. In addition, although the same target proteins may be present in the library, different binding conditions used in each screen could also result in unique subsets of proteins that are recognized by the bait molecule. We compared the molecules contained in these three defined libraries used for LNX1 interaction screens and found that on average about half the proteins in a given library are found in one or both of the other two libraries. When we searched for proteins that were recognized by LNX1 in any one of these screens, we found that there was greater potential for overlap than was actually observed. Specifically, 26 of the 43 molecules identified in the yeast two-hybrid screen [42] were contained in the protein array library (this study) while none of the 43 molecules were found in the peptide library [37]. This unrealized potential for overlap in the screen results illustrates the varying ability of the same bait molecule to bind to the same target molecules under different screen conditions.

Previously, LNX1 PDZ1 has been shown to bind the intracellular proteins SKIP [26], Src [27] and Numb [12]. The ability of PDZ1 to recognize only the isoforms of Numb that are ubiquitinated by LNX1 [12] suggests that PDZ1 may play an important role in substrate recognition or orientation relative to the E2-bound RING domain of LNX1. We found that LNX1 PDZ1 is able to recognize two novel binding partners, KCNA4 and PLEKHG5. KCNA4 is a shaker-type voltage-gated K^+^ channel [49], [50]. To our knowledge, this is the first time that LNX1 PDZ1 has been verified to bind a transmembrane protein and the first time that any PDZ domain of LNX1 has been found to interact with an ion channel protein. The carboxy terminal tails of both KCNA4 and PLEKHG5 fit a traditional PDZ binding motif (S/T-X-V) whereas SKIP, Src and Numb do not. As LNX1 PDZ1 also binds to an internal site in the Numb PTB domain [12], it is possible that PDZ1 recognizes both canonical PDZ domain binding motifs as well as internal sites on other protein targets.

LNX1 PDZ2 has been found to bind both intracellular and transmembrane targets [13], [14], [16] and has been predicted to bind a number of other proteins [37], [43]. Here we confirmed predicted interactions with Tyk2 and PAK6, and identified a novel interaction partner, PKC-alpha1. Tyk2, PAK6 and PKC-alpha1 are all protein kinases. TYK2 is a member of the Janus kinase (JAK) family of cytoplasmic protein tyrosine kinase [51], [52] while PAK6 and PKC-alpha1 are serine/threonine kinases associated with a wide variety of cellular processes including signal transduction, cell proliferation and apoptosis [53]–[59]. The ability of LNX1 PDZ2 to bind a wide variety of cytoplasmic and transmembrane proteins including protein kinases suggests that this domain plays a critical role in recruiting components to the LNX1 signal transduction complex. The screen of a random peptide library using LNX1 PDZ2 predicted that PDZ2 would select proteins with a cysteine residue in the carboxy terminal position [37] and a recent random peptide library screen also revealed that PDZ domains from several other proteins select binding motifs with a carboxy terminal cysteine [36]. Here we confirmed by coimmunoprecipitation and GST pull down experiments that LNX1 PDZ2 is able to bind Tyk2 and PAK6, two proteins that contain a carboxy terminal cysteine residue.

The first published binding partner for LNX1 PDZ3 was the proto-oncogene c-Src, a cytoplasmic protein tyrosine kinase [28]. Although the interaction between LNX1 and Src was identified through PDZ3, the authors found that PDZ1 is sufficient for the interaction with Src. These authors also demonstrated that LNX1 is phosphorylated by Src and that Src is ubiquitinated by LNX1. These findings support our previous findings that PDZ1 is important for recognition of ubiquitination targets [12]. Here we identified an interaction between LNX1 PDZ3 and PLEKHG5. Similar to Src, PLEKHG5 is also able to bind to PDZ1. Therefore, PDZ1 and PDZ3 may bind to similar targets potentially increasing LNX1 substrate avidity.

Previously, no ligands have been identified for LNX1 PDZ4. Here we identify two protein ligands, PKC-alpha1 and PAK6, as well as multiple PIPs as ligands for PDZ4. Both PKC-alpha1 and PAK6 also interact with PDZ2. Only PDZ4 was able to bind to PIPs, suggesting that an important function of PDZ4 is to interact with membrane phospholipids that may influence LNX1 subcellular localization. Interestingly, a similar binding specificity has been observed for PTP-BL (Protein Tyrosine Phosphatase - Basophil Like) PDZ5, i.e., very few protein interactors and the ability to bind phospholipids [60].

Our work has shown that each PDZ domain of LNX1 contributes a specific element to the overall function of LNX1 as a signal transduction complex scaffold. We also considered that as components of a scaffold protein, individual PDZ domains of LNX may interact with distinct elements of a single pathway. Analysis of the list of 220 LNX interacting proteins using Reactome Pathway Analysis (http://www.reactome.org) highlighted two pathways that are statistically over represented. This analysis suggests a role for LNX1 in tight junction organization through association with claudin family members as well as a potential role for LNX1 in GAP junction trafficking and regulation. Future work to identify the specific molecules present in the LNX1 multi-protein signalling complex in different cell types will be needed to elucidate LNX1 functions.

## Supporting Information

Figure S1 **Coomassie stained SDS PAGE gel showing LNX1 GST-PDZ domain fusions used to probe ProtoArrays® before and after labelling with Alexa Fluor 647.**
(TIFF)

Figure S2 **Comparison of the amino acid sequences of 35 PDZ domains, 29 type I domains as classified by Tonikian et al.** [**36**] **, four PDZ domains shown to bind peptides with cysteine in the 0 position including PDZ-RhoGEF** [**39**] **, PTP-BL** [**40**] **and SITAC** [**41**] **, as well as LNX1 PDZ2 and LNX2 PDZ2. Cys-peptide binding PDZ domains are highlighted in blue.** ClustalW generated a guidetree from the distance matrix of the alignments, visualized using Dendroscope [61].
(TIFF)

Table S1 **List of proteins that was positive for LNX1 PDZ domain binding on both ProtoArray® slides.**
(XLS)

Table S2 **Comprehensive list of LNX1 PDZ domain interacting proteins assembled from ProtoArray® and yeast two-hybrid screens as well as those previously identified in three separate high-throughput screens** [**37]**, [42], [43****] **.**
(XLS)

Table S3 **The carboxy terminal sequences of confirmed LNX PDZ domain interacting proteins was used to search the complete list of interactors in Table S2.** Twenty-eight proteins with similar carboxy terminal sequences were identified.
(XLS)
