# Supplementary figures and images for: Biochemical and Computational Analysis Of LNX1 Interacting Proteins
Source: PLoS One. 2011 Nov 8;6(11):e26248. doi: 10.1371/journal.pone.0026248 (PMC3210812; doi:10.1371/journal.pone.0026248)

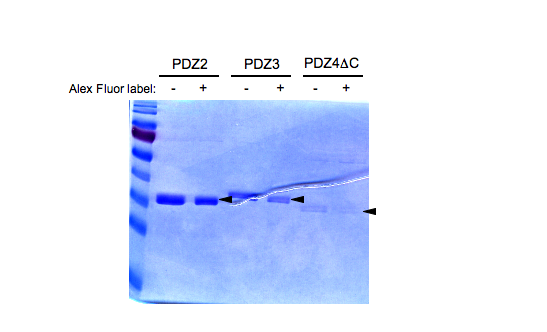

Supplement: Figure S1 — Coomassie stained SDS PAGE gel showing LNX1 GST-PDZ domain fusions used to probe ProtoArrays® before and after labelling with Alexa Fluor 647. (TIFF) [file pone.0026248.s001.tif]

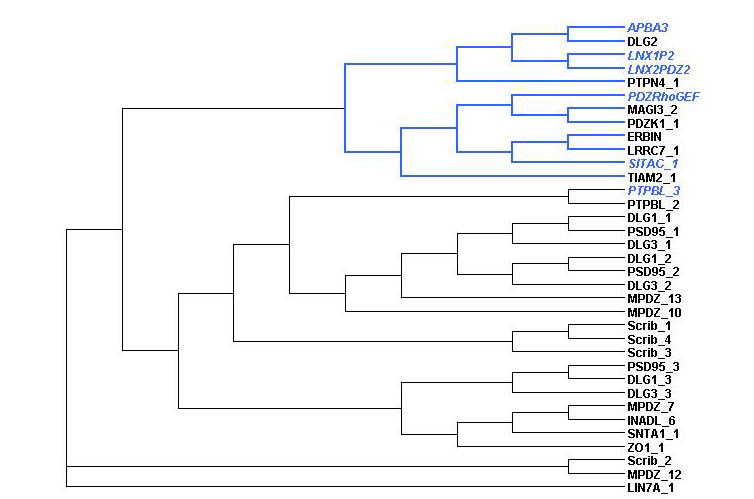

Supplement: Figure S2 — Comparison of the amino acid sequences of 35 PDZ domains, 29 type I domains as classified by Tonikian et al. [36] , four PDZ domains shown to bind peptides with cysteine in the 0 position including PDZ-RhoGEF [39] , PTP-BL [40] and SITAC [41] , as well as LNX1 PDZ2 and LNX2 PDZ2. Cys-peptide binding PDZ domains are highlighted in blue. ClustalW generated a guidetree from the distance matrix of the alignments, visualized using Dendroscope [61]. (TIFF) [file pone.0026248.s002.tif]
